# Emerging Functions of RANKL in Lymphoid Tissues

**DOI:** 10.3389/fimmu.2012.00261

**Published:** 2012-09-03

**Authors:** Christopher G. Mueller, Estelle Hess

**Affiliations:** ^1^CNRS, Laboratory of Therapeutic Immunology and Chemistry, Institut de Biologie Moléculaire et Cellulaire, University of StrasbourgStrasbourg, France; ^2^Unité de Recherche en Biologie Moléculaire, Facultés Universitaires Notre-Dame de la Paix NamurNamur, Belgium

**Keywords:** TRANCE, TNFSF11, OPG, lymphoid organs, lymph node, stroma, LTi, LTo

## Abstract

The tumor necrosis factor superfamily (TNFSF) members play pivotal roles in embryonic development of lymphoid tissue and their homeostasis. RANKL (Receptor activator of NF-κB ligand, also called TRANCE, TNFSF11) is recognized as an important player in bone homeostasis and lymphoid tissue formation. In its absence bone mass control is deregulated and lymph nodes fail to develop. While its function in bone is well described, there is still little functional insight into the action of RANKL in lymphoid tissue development and homeostasis. Here we provide an overview of the known functions of RANKL, its signaling receptor RANK and its decoy receptor OPG from the perspective of lymphoid tissue development and immune activation in the mouse. Expressed by the hematopoietic lymphoid tissue inducing (LTi) cells and the mesenchymal lymphoid tissue organizer (LTo) cells, RANKL was shown to stimulate Lymphotoxin (LT) expression and to be implicated in LTi cell accumulation. Our recent finding that RANKL also triggers proliferation of adult lymph node stroma suggests that RANKL may furthermore directly activate LTo cells. Beyond bone, the RANKL-RANK-OPG triad plays important roles in immunobiology that are waiting to be unraveled.

## Introduction

Tumor necrosis factor (TNF) and Lymphotoxin (LT) were identified as the first members of a large family, now called the TNF-superfamily (SF). Not surprisingly, the receptors for these proteins also constitute a SF with sequence homology, named TNF Receptor (TNFR) SF. A hallmark of these ligand-receptor pairs lies in a threefold symmetry, where by the oligomeric binding arrangement amplifies their avidity and introduces flexibility. Further complexity arises through different partner affinities and generation of soluble ligand and receptor forms (Bodmer et al., 2002). RANKL (TNFSF11) is the ligand of two receptors, RANK (TNFRSF11a) and OPG (TNFRSF11b). OPG (osteoprotegerin) was the first of this protein triad to be discovered (Simonet et al., 1997) in a search for an inhibitor of osteoclastogenesis (Tsuda et al., 1997). OPG-ligand was then isolated and cloned using OPG as bait (Lacey et al., 1998; Yasuda et al., 1998). OPG-ligand turned out to be identical to TRANCE (TNF-related activation induced cytokine), cloned during a search for apoptosis-regulatory genes in T cells (Wong et al., 1997b), and RANKL (Receptor activator of NF-κB) identified as the ligand for RANK that had attracted attention for its homology to CD40 (Anderson et al., 1997). The affinity of RANKL for OPG is 1000-fold higher than for RANK (Nakagawa et al., 1998), which is dependent on the ability of OPG to homodimerize (Schneeweis et al., 2005). OPG is also a ligand for TNF-related apoptosis-inducing ligand (TRAIL; Emery et al., 1998), however, its affinity for TRAIL is 10,000 times less compared to RANKL (Body et al., 2006) questioning the *in vivo* relevance of OPG-TRAIL interaction. There is now an emerging consensus to refer to the receptor as RANK and, as a consequence and for simplicity, its ligand is called RANKL. The acronym OPG has remained in use.

The discovery of RANK, RANKL, and OPG in bone and the immune system raises the question of its evolutionary origins. The genes arose simultaneously during ontogeny of bony fish as evidenced by gene sequence identification and presence of resorption and remodeling activity of vertebrate mineralized tissue (Witten and Huysseune, 2009). They therefore postdate the formation of the primordial immune system comprising a primitive thymus and lymphoid structures associated with exposed sites. However, they preceded the development of lymph nodes (LNs) and germinal centers arising in amphibians and the emergence of LT β receptor, a key molecule in lymphoid development (Glenney and Wiens, 2007; see below). It is therefore likely that the RANK-RANKL-OPG protein triad was co-opted by the advanced immune system for higher order structure together with an efficient regulation of immune cell output from the bone marrow before genesis of LT β receptor-regulated lymphoid tissues.

RANKL is a type-II transmembrane protein but can also exist in a soluble form by ectodomain shedding and alternative splicing (Ikeda et al., 2001; Hikita et al., 2006; Baud’huin et al., 2007). OPG comprises two C-terminal regions homologous to death domains of TNFR1 or TRAIL receptor, which were found to be functional when OPG was expressed with a transmembrane sequence (Yamaguchi et al., 1998). Natural OPG is unlikely to transmit signals because it misses the transmembrane sequence and is secreted (Simonet et al., 1997). RANK comprises a transmembrane region and a large cytoplasmic domain. Upon interaction with the RANKL trimer RANK undergoes homotrimerization and activates recruitment of TNFR-associated factors (TRAFs; Galibert et al., 1998; Wada et al., 2006). RANK binds five of the six known TRAF-proteins but TRAF6 seems particularly important for RANK signaling, because TRAF6^−/−^ mice present similar phenotypes as *Rank*^−/−^ mice (Naito et al., 1999). RANK signaling cascades were mostly deciphered in the myeloid lineage and include the canonical and the non-canonical NF-κB pathways (Raju et al., 2011). In mammary glands RANK-activation intersects with proliferative cues through cyclin D1, Id2, and Id4 (Schramek et al., 2011). RANK was recently found to play a role in mammary and in hair follicle epithelial stem cell activation (Schramek et al., 2010; Duheron et al., 2011) and to induce intestinal microfold cells (M cells) differentiation via the Ets transcription factor Spi-B (Kanaya et al., 2012).

## RANKL in Bone and Hematopoiesis

**Figure 1:**
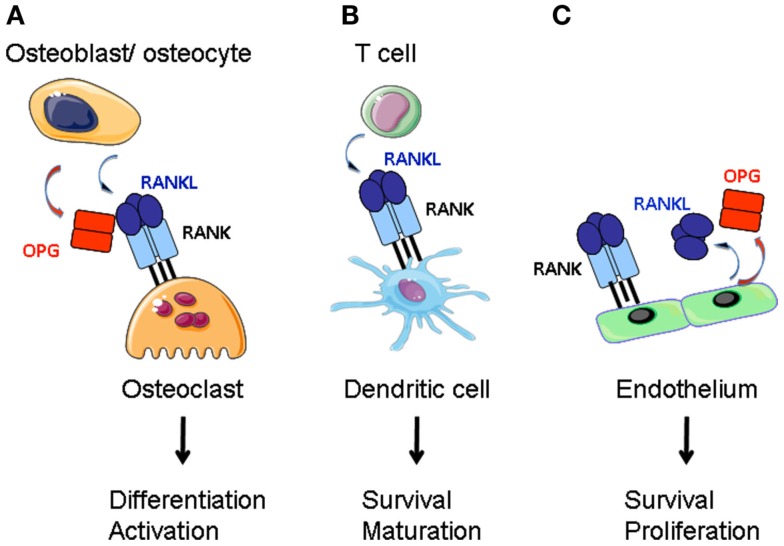
**RANKL function in the bone, the adaptive immune system and the vascular system**. **(A)** RANKL produced by bone marrow stroma (osteoblasts and osteocytes) directs the differentiation of preosteoclasts of the myeloid lineage into mature osteoclasts. Stroma-derived OPG negatively regulates RANKL-RANK interaction. **(B)** Activated T cells express RANKL that stimulates dendritic cell survival and maturation. **(C)** Endothelium is a source for RANKL and OPG. RANK-activation of endothelial cells supports cell survival and promotes angiogenesis.

A number of reviews have been published on the role of these proteins in regulating bone mass (Suda et al., 1999; Walsh and Choi, 2003; Baud’huin et al., 2007; Leibbrandt and Penninger, 2010). *Rankl*^−/−^ and *Rank*^−/−^ mice present osteopetrosis and lack of teeth (Dougall et al., 1999; Kong et al., 1999), whereas *Opg*^−/−^ animals exhibit osteoporosis (Bucay et al., 1998; Mizuno et al., 1998; Yun et al., 2001). RANK activates the differentiation of bone matrix degrading osteoclasts (OCL) from myeloid precursor cells (Yasuda et al., 1998; Hsu et al., 1999; Figure 1). RANKL and OPG are synthesized by the bone mesenchymal lineage and are under inflammatory and hormonal control (Udagawa et al., 1999; Takeda et al., 2003; Nakashima et al., 2011; Xiong et al., 2011). Another source of RANKL is activated T cells that can cause abnormal bone resorption by triggering osteoclastogenesis (Takayanagi et al., 2000; Sato et al., 2006).

T cell lymphopoiesis itself underlies RANK regulation as *Rank*^−/−^ mice present a block in the progression to CD4^−^CD8^−^CD44^−^CD25^−^ thymocytes (Kong et al., 1999). Recently it was shown that also Vγ5^+^ T cells are under RANK regulatory action (Roberts et al., 2012). In fact RANK signaling is a key event in the early stages of medullary thymic epithelial cell (mTEC) formation, and its cooperation with LT and CD40 signals is required to establish a fully developed medullary microenvironment (Rossi et al., 2007; Akiyama et al., 2008; Hikosaka et al., 2008; Mouri et al., 2011). mTEC play a crucial role in self-tolerance by eliminating self-reactive αβT cells and by regulating the early production of γδT cells. Thymic CD3^−^CD4^+^ lymphoid tissue inducing (LTi) cells and Vγ5^+^ thymocytes as well as later arising CD4^+^CD8^−^ single positive thymocytes and ΓδT cells are equipped with RANKL (Rossi et al., 2007; Hikosaka et al., 2008; Roberts et al., 2012). RANK and OPG are expressed by mTECs (Hikosaka et al., 2008). Animals defective in RANK signaling also display abnormal B cell hematopoiesis and hypogammaglobulinemia (Dougall et al., 1999; Kong et al., 1999). Although B cells express RANK, in particular in response to activation (Yun et al., 1998; Perlot and Penninger, 2012), a B cell-specific RANK knock-out mouse does not reproduce this phenotype (Perlot and Penninger, 2012), suggesting that the defect lies in bone marrow or splenic stroma.

## RANKL in Early Stages of Secondary Lymphoid Organ Development

**Figure 2:**
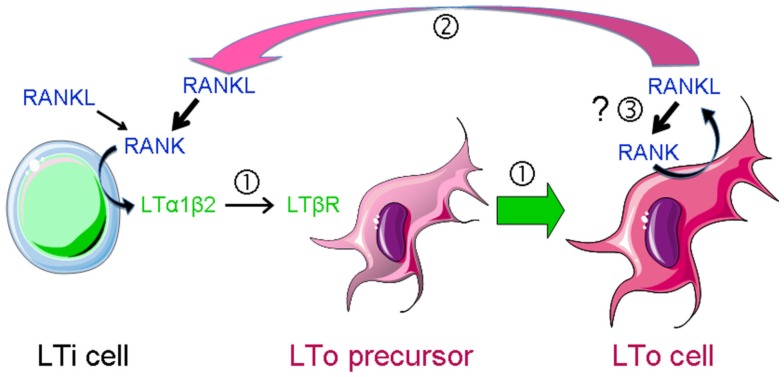
**RANKL-dependent amplification loops in SLO development**. SLO development during embryogenesis is initiated by the recruitment of lymphoid tissue inducer (LTi) cells to a rudimentary organ anlage composed of mesenchymal lymphoid tissue organizer (LTo) cell progenitors. This step is followed by a cross-talk between LTi cells that express RANKL, RANK, and LTαβ and LTo cells that carry LTβR 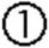. Engagement of the latter induces the maturation of LTo cells 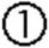 to express RANKL and chemokines that attract larger numbers of LTi cells that upon clustering with LTo cells initiate LN organization. LTo cells also express RANKL. The current model of LN development indicates that a positive feedback loop takes place between LTo and LTi cells via RANKL-RANK and LTαβ-LTβR amplifying the early stages of LN development 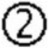. If LTo cells expressed also RANK, a second (autocrine) loop may occur, leading to a direct activation of LTo cells 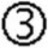.

RANK and RANKL-deficient animals display a complete absence of LNs, defects in Peyer’s patches (PPs) and cryptopatches (CPs) and abnormalities of the spleen (Dougall et al., 1999; Kong et al., 1999; Kim et al., 2000; Knoop et al., 2011; Perlot and Penninger, 2012). Therefore the RANK-RANKL-OPG axis shares with the LT and TNFα pathways the control of molecular and cellular processes determinant in secondary lymphoid organ (SLO) development (Tumanov et al., 2003; Fritz and Gommerman, 2010). SLO formation is initiated around embryonic day (E) 15 with the recruitment of the hematopoietic LTi cells to a rudimentary organ anlage composed of mesenchymal and endothelial stroma (White et al., 2007; Vondenhoff et al., 2009; Benezech et al., 2011). The recruitment process is dependent on the chemokine CXCL13 produced by precursors of lymphoid tissue organizer (LTo) cells stimulated by neuronal production of retinoic acid (van de Pavert et al., 2009). This step is followed by a cross-talk between LTi cells that express RANK, RANKL, and LT, and LTo precursors that carry the LT receptor LTβR. LTβR engagement induces LTo cells to express RANKL and chemokines to attract larger numbers of LTi cells that upon clustering with LTo cells initiate LN organization (Cupedo and Mebius, 2005; Koning and Mebius, 2011; Figure 2).

In view of the finding that LTi cell recruitment is LT independent (Eberl et al., 2004; White et al., 2007; Vondenhoff et al., 2009) the question arises whether LTi cell accumulation is regulated by RANK. Both *Rankl*^−/−^ and *Ltα*^−/−^ mice have lower number of LTi cells in mesenteric LNs of newborn mice (Kim et al., 2000). TRAF6^−/−^ mice display fewer LTi cells in mesenteric LNs at E 17.5 but not at E15.5 (Yoshida et al., 2002). Administration of RANK-Fc antagonist led to a partial reduction in LTi cells, with a more prominent effect in mesenteric LNs (Eberl et al., 2004). Therefore, although current data do not unambiguously support RANKL as a direct regulator of LTi cell numbers, they sustain the concept that RANKL is instrumental for LTi cell accumulation. Of note, RANK signaling mediators include Id2 (Kim et al., 2011), a factor indispensable for LTi cell formation (Yokota et al., 1999). It is hence plausible that Id2 is implicated in RANK regulation of LTi cell numbers and function.

In the current model of LN development a positive feedback loop takes place between LTi and LTo cells (Figure 2). RANK signaling in LTi cells increases the expression of LT that upon binding to LTβR of LTo cells induces RANKL production, thus amplifying the early stages of LN development (Yoshida et al., 2002; Koning and Mebius, 2011; Roozendaal and Mebius, 2011). In support for such a feedback loop is the observation that RANKL expression is up to 10-fold higher in LTo cells than in LTi cells (Sugiyama et al., 2012). However, it is unlikely that the function of RANKL in SLO development is limited to the induction of LT by LTi cells and the creation of the amplification loop. Firstly, LT is upregulated by a number of other factors, such as IL-7, TNFα, and CXCL13 (Ansel et al., 2002). Indeed PPs are LT-dependent but develop in *Rankl*^−/−^ mice. Second, mucosal LNs develop in LTβ^−/−^ mice but not in *Rankl*^−*/*−^ mice (Alimzhanov et al., 1997; Koni et al., 1997). Finally, administration of an LTβR-agonistic antibody to *Rankl*^−/−^ embryos cannot rescue LN genesis (Kim et al., 2000).

LTβR signaling in mTECs induces RANK expression (Mouri et al., 2011). We have recently shown that RANK is expressed in adult LN stroma and induces hyperproliferation of reticular fibroblastic and vascular cells (Hess et al., 2012). Expression of both RANK and RANKL by LTo cells may therefore trigger a second (autocrine) loop leading to a direct activation of these cells (Figure 2). In addition, vascular endothelial cells express RANK, RANKL, and OPG that together regulate angiogenesis (Kim et al., 2003; Benslimane-Ahmim et al., 2011; Sugiyama et al., 2012; Figure 1).

## RANKL and SLO Growth

A number of observations support a role of RANKL in SLO growth. The LNs that developed after neutralization of RANK signaling in embryos were smaller (Eberl et al., 2004; Sugiyama et al., 2012). PPs, CPs, and isolated lymphoid follicles (ILFs) were reduced in size in *Rankl*^−/−^ mice (Knoop et al., 2011), and postnatal RANKL overproduction led to massive LN hyperplasia (Hess et al., 2012). Recruitment of immune cells and stromal cell division stand out among possible regulatory mechanisms of SLO size. Lymphocyte recruitment into SLO anlage coincides with maturation of LTo cells to produce high levels of chemokines and cell adhesion molecules (Honda et al., 2001; Finke et al., 2002; Luther et al., 2003; Cupedo et al., 2004b; White et al., 2007; Benezech et al., 2011). In mice with postnatal LN hyperplasia RANKL upregulates CXCL13, CCL19, MAdCAM-1, and VCAM-1 gene transcription in adult fibroblastic reticular cells (FRCs) and vascular cells (Hess et al., 2012). Therefore, RANKL could directly boost immune cell accumulation by increased chemokine and adhesion factor output. While recruitment of stromal precursors from surrounding tissue or bone cannot be excluded, CD45-negative cells label for the cell division marker Ki-67 as early as E16 (Eberl et al., 2004; White et al., 2007). Although this proliferation appears to be dependent on LT (White et al., 2007), it is unclear whether the proliferating cells are endothelial cells, precursor or mature LTo cells. We have found that RANKL stimulates FRC and endothelial cell proliferation (Hess et al., 2012). Further support for a functionally important role of RANKL in cell proliferation stems from findings that thymic mTECs and skin keratinocyte cell growth is accelerated in response to RANK stimulation (Hikosaka et al., 2008; Duheron et al., 2011).

## Toward a Role of RANKL in B Cell Recruitment and Follicle Organization

B cell recruitment and organization into follicles occur in a CXCL13-dependent manner at later stages of SLO formation (Ansel et al., 2000; Cupedo et al., 2004a). B cell follicular dendritic cells (FDCs) and the recently identified marginal reticular cells (MRCs), both mesenchymal cell types, are the main producers of this chemokine (Ansel et al., 2000; Katakai et al., 2008). First supportive evidence for a role of RANKL in B cell recruitment and organization was provided after the rescue of LNs by exogenous IL-7 in TRAF6^−/−^ mice: it was noted that in these LNs B cells and FDCs were absent (Yoshida et al., 2002). However, because TRAF6 is also a signaling component for TNFR, a critical receptor for FDC formation (Rennert et al., 1998; Endres et al., 1999), a role of RANK signaling in B cell recruitment cannot be directly invoked. More direct evidence was provided by administration of a RANKL-neutralizing antibody to embryos. This resulted in reduced LN B cells numbers, misplaced FDCs, and reduced VCAM-1 staining (Sugiyama et al., 2012). In addition, Knoop et al. (2011) noted an absence of B cells in small intestine CPs of *Rankl ^−/−^* mice and observed that most stromal cells in the B cell compartment lacked VCAM-1 and CXCL13 expression. Finally, postnatal RANKL overexpression resulted in an increase in small but clearly defined B cell follicles, which all comprised FDCs (Hess et al., 2012). Three possible scenarios can be advanced to explain these phenomena: (i) RANKL increases the bone marrow B cell output, (ii) CXCL13 production by FDCs and/or MRCs is under RANKL positive control, (iii) RANK-signaling promotes MRC and/or FDC differentiation. Although the first scenario appears plausible in view of the known action of RANKL in the bone, so far, there is no experimental support for this idea. The rise in LN B cell numbers in response to RANKL overproduction is not accompanied by an expansion in splenic transitional B cell subsets (Hess et al., 2012). As for the second model, there is evidence that RANKL upregulates CXCL13 gene transcription in FRCs, however the level of induction was low (Hess et al., 2012). Lastly, reduction of VCAM-1 expression by FDCs is indicative of a requirement of RANK-signaling for terminal differentiation of FDCs. In keeping with this idea, reduced CXCL13 expression by FDCs could be the consequence of FDC dysfunction. It is intriguing that MRCs, which have been proposed to function as FDC precursors, express RANKL (Katakai et al., 2008). Cells that bear resemblance to LN MRCs have also been found in the spleen, PP, and ILF on the grounds of RANKL expression and independence of LTβR signaling (Taylor et al., 2007; Katakai et al., 2008). The polarized expression of RANKL beneath the follicle-associated epithelium may be necessary to focus its activity of inducing differentiation of intestinal M cells, cells specialized in the transport of antigen to the underlying lymphoid tissue (Knoop et al., 2009). It is plausible that RANKL jointly regulates FDC differentiation and (native) antigen access.

## RANKL and the Adaptive Immune Response

Activated CD4 and CD8 T cells express surface and soluble RANKL (Josien et al., 1999; Wang et al., 2002; Figure 1). Dendritic cells are of the same lineage as OCL and express RANK (Anderson et al., 1997). RANKL confers to DCs better survival with more notable effects on *in vitro* generated DCs and in combination with other TNFSF members (Wong et al., 1997a; Dougall et al., 1999; Josien et al., 2000; Williamson et al., 2002). Stimulation of DCs results in production of pro-inflammatory cytokines IL-6, IL-1β, and T cell differentiation factors IL-12, IL-15 (Josien et al., 1999). However, other reports have noted anti-inflammatory activity for RANKL. In a model of oral tolerance, RANKL stimulation of DCs has been associated with tolerance induction (Williamson et al., 2002). An anti-inflammatory effect was also noted for RANKL-stimulated Langerhans cells and macrophages (Maruyama et al., 2006; Yoshiki et al., 2009). This discrepancy may be due to low RANK expression level in immature DCs; its expression being upregulated in response to Toll-like receptor (TLR) ligands or inflammatory cytokines (Hochweller and Anderton, 2005). Another explanation could be a redundancy with other TNFRSF members such as its close homolog CD40 (Bachmann et al., 1999). Alternatively, activated DCs express OPG, thus inhibiting RANKL (Schoppet et al., 2007). Except for a reduction in Langerhans cell numbers (Barbaroux et al., 2008), there is little experimental support that the RANK-RANKL-OPG triad controls DC development *in vivo* (Dougall et al., 1999).

Th17 T cells represent an important osteoclastogenic T cell type by robust RANKL production and activation of RANKL release by mesenchymal cells (Sato et al., 2006). This T cell type is of particular importance in progressive periodontitis, a dental disease characterized by destruction of alveolar bone with high prevalence of bacteria such as *Porphyromonas gingivalis* (Kajiya et al., 2010). In this disease, periodontal ligament fibroblasts are an important source of RANKL when stimulated by microbial products including TLR ligands. TLRs are also expressed by osteoclast precursors and OCL and their stimulation promotes osteoclastogenesis and maturation of OCL. Interestingly, gingival Langerin-expressing DCs have recently been shown to control inflammation in *P. gingivalis*-induced periodontitis and therefore reduce alveolar bone loss (Arizon et al., 2012). It is yet unclear whether this occurs via a direct RANKL-induced DC anti-inflammatory activity.

## Conclusion

The RANK-RANKL-OPG axis plays a recognized role in bone homeostasis through the regulation of osteoclastogenesis. It is also implicated in SLO development and regulation of the immune response. There are many incentives to answer remaining questions. In addition to a restless curiosity of the researcher, tertiary lymphoid tissues that arise in inflamed tissue rely on similar if not identical cellular dialogs as those found in SLO development. Defining RANKL function in lymphoid tissue development will open new therapeutic avenues to treat inflammatory diseases and provide new strategies for vaccine development.

## Conflict of Interest Statement

The authors declare that the research was conducted in the absence of any commercial or financial relationships that could be construed as a potential conflict of interest.
